# Laparoscopic Roux-en-Y feeding jejunostomy as a long-term solution for severe feeding problems in children

**DOI:** 10.1007/s00431-022-04705-3

**Published:** 2022-11-18

**Authors:** H. van Braak, R. R. Gorter, M. P. van Wijk, J. R. de Jong

**Affiliations:** 1 Department of Pediatric Surgery, Amsterdam University Medical Center, Noord-Holland, Meibergdreef 9, 1105 AZ Amsterdam, The Netherlands; 2 Department of Pediatric Gastroenterology, Amsterdam University Medical Center, Noord-Holland, Meibergdreef 9, 1105 AZ Amsterdam, The Netherlands

**Keywords:** Gastric emptying, Laparoscopic jejunostomy, Roux-en-Y, Children, Pediatric, Feeding problems

## Abstract

Enteral feeding is a common problem in children with gastric emptying disorders. Traditional feeding methods in these patients often show a high rate of complications and maintenance issues. Laparoscopic Roux-en-Y feeding jejunostomy (LRFJ) has been described in a few patients as a minimal invasive option for enteral access in these children. The aim of this study is to evaluate the outcomes of the LRFJ procedure in our tertiary referral center. We conducted a retrospective case-series including all patients, aged 0–18 years old, that underwent a LFRJ procedure between August 2011 and December 2020 for the indication of oral feeding intolerance due to delayed gastric emptying. Outcomes evaluated were complications (short and long term) and parenteral satisfaction. In total, 12 children were identified that underwent LRFJ for the indication of oral feeding intolerance due to delayed gastric emptying. A total of 16 complications were noted in 8/12 patients (67%). Severity classified by Clavien-Dindo were grade I (*n* = 13), grade II (*n* = 1), and grade IIIB (*n* = 2). In 11/12 patients, parents were satisfied with the results.

*Conclusions*: Although minor complications after LRFJ are common in our patients, this technique is a safe solution in patients with gastric emptying disorders leading to a definitive method of enteral feeding and high parenteral satisfaction.

**Table:** 

**What is Known:** *• Traditional tube feeding in children (duodenal, PEG-J-tubes) with severe delayed gastric emptying can be challenging with a high rate of complications and maintenance issues.* *• Open loop jejunostomy and Roux-en-Y jejunostomy are alternative, permanent methods of feeding but either invasive or are accompanied by severe complications. Little is known in the literature about laparoscopic Roux-en-Y feeding jejunostomy.*	
**What is New:** *• Laparoscopic Roux-en-Y feeding jejunostomy is a permanent, safe and minimal invasive alternative option for enteral feeding in children with severe delayed gastric emptying..*

**What is Known:**

*• Traditional tube feeding in children (duodenal, PEG-J-tubes) with severe delayed gastric emptying can be challenging with a high rate of complications and maintenance issues.*

*• Open loop jejunostomy and Roux-en-Y jejunostomy are alternative, permanent methods of feeding but either invasive or are accompanied by severe complications. Little is known in the literature about laparoscopic Roux-en-Y feeding jejunostomy.*

**What is New:**

*• Laparoscopic Roux-en-Y feeding jejunostomy is a permanent, safe and minimal invasive alternative option for enteral feeding in children with severe delayed gastric emptying..*

## Introduction

Feeding management in children with severe delayed gastric emptying can be challenging. Delayed gastric emptying is a rare disease that has an incidence of 4:100 000 in the overall pediatric population [1] and a high prevalence in children with mitochondrial diseases [2, 3]. Initial treatment of a child with severe delayed gastric emptying consists of pharmacological interventions (stimulation of gastric peristalsis) often in combination with continuous gastric feeds. In case of failure, the next step consists of the placement of nasoduodenal or nasojejunal feeding tubes or percutaneous endoscopic gastro-jejunal tubes (PEG-J-tubes). Both options, however, are associated with a high rate of complications and maintenance issues (dislodgement and displacement (7–13%), leakage (6–13%), nasal irritation and opposition from the patients, pneumoperitoneum (0.7–7%), infection (local 5.6–12%, systemic 3.5%), and hypergranulation (10.3%)) [4–9]. In addition, in case of displacement, sedation or general anesthesia is often required as spontaneous repositioning is often not possible. Additional techniques, like magnetic guidance or fluoroscopy to position the tubes, are frequently needed. This increases the burden for patients (due to frequent exposure of the child to anesthesia and radiation) and parents (due to frequent hospital visits) [10, 11].

An alternative to achieve full enteral feeding in these children is a surgically placed jejunostomy. Two main surgical techniques have been described in the pediatric population. In the classic way (or so-called loop jejunostomy), the jejunum is stitched to the abdominal wall and an opening is created through the abdominal wall through which a tube is introduced into the jejunum [12]. In the available literature however, severe complications such as leakage of enteric contents, bowel obstruction, and difficulties in replacing a dislodged tube have all been described [13–15]. The other technique is the so-called Roux-en-Y-jejunostomy. This original Roux-en-Y procedure, first described by Maydl in 1888, was originally done using an open procedure where the end of the Roux limb was brought up through the abdominal wall and anastomosed to the skin creating a permanent stoma [16]. Multiple modifications of this techniques have been described in the literature [17, 18]. A Roux-en-Y-jejunostomy can also be performed laparoscopically, laparoscopic-assisted Roux-en-Y feeding jejunostomy, as described in one study with good results in children [19]. In our clinic, we prefer this last method. As demonstrated by a recent systematic review evaluating the outcomes of Roux-en-Y jejunostomy in the pediatric population, more data regarding the outcomes of LRFJ are lacking in current literature. All but one of the included studies evaluated the outcomes in children undergoing open Roux-en-Y jejunostomy [20].

This study is aimed at evaluating the outcomes of the LRFJ procedure in our tertiary referral center in order to contribute to the literature on the safety and effectiveness of the procedure.

## Materials and methods

### Study design and patient selection

We conducted a retrospective case-series including all patients, aged 0–18 years old, that underwent a LFRJ procedure between August 2011 and December 2020 for the indication of oral feeding intolerance due to delayed gastric emptying. This study was performed in a tertiary referral center for pediatric surgery in the Netherlands. The diagnosis of delayed gastric emptying was made on clinical symptoms as reference values for scans were not applicable on pediatric patients at the time of this study. All patients received enteral feeding using nasoduodenal tubes or PEG(-J)-tubes prior to the surgical procedure. After failure of traditional treatment (feeding with pharmacological interventions) and a trajectory of enteral feeding (using nasoduodenal or PEG(-J)-tubes) troubled by complications and maintenance issues, patients were discussed in a multidisciplinary team meeting in which pediatric surgeons, pediatric gastroenterologists, (and if needed radiologist and dietitians) participated. In all cases, the combination of an extensive history of oral feeding intolerance, tube feeding, and earlier procedures troubled by complications and maintenance issues led to the decision to perform LRFJ.

Patients that underwent other procedures (e.g. loop jejunostomy) were excluded from this study.

### Technique

**Fig. 1:**
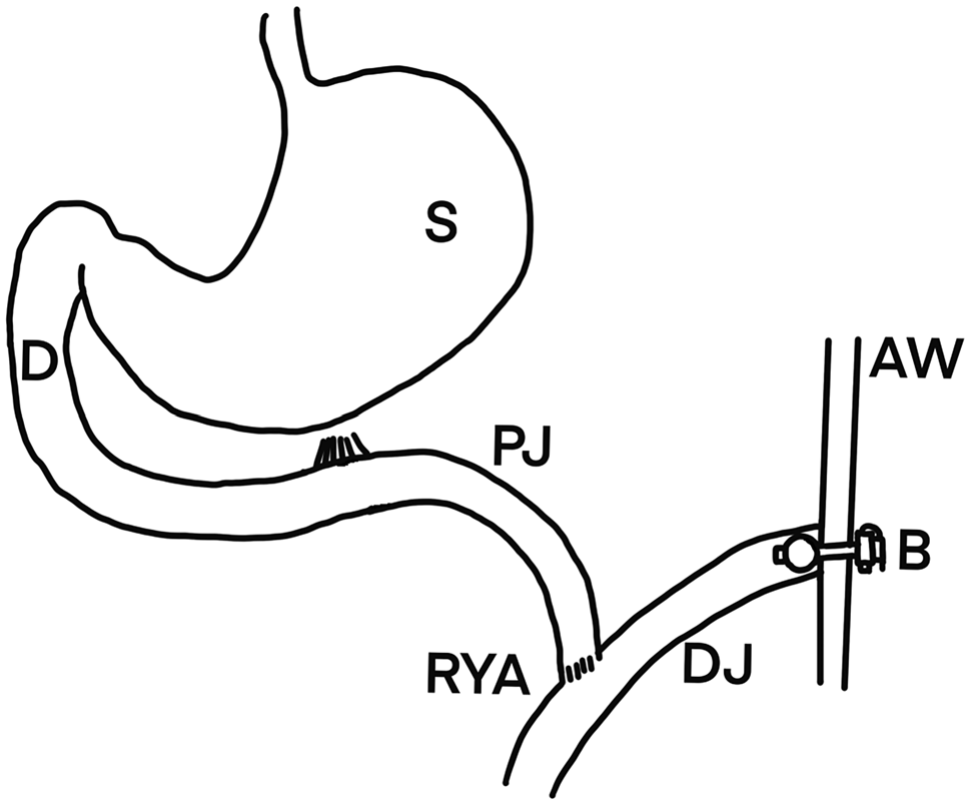
Illustration of the used surgical procedure. The afferent end of the jejunum is pulled against the abdominal wall and a gastrostomy tube is inserted in it. An end-to-side anastomosis is made between the afferent and efferent part of the jejunum. AW = abdominal wall, B = button, D = duodenum, DJ = distal jejunum, PJ = proximal jejunum, RYA = Roux-en-Y anastomosis

The surgical procedure was done by two pediatric surgeons specialized in complex colorectal surgery including motility disorders. They use a slightly modified technique derived from the procedure as described by Weidner [21]. This technique is illustrated in Fig. 1. Patients are positioned in a supine position on the table in reverse Trendelenburg and tilted to the right. Three trocars are placed, one at the level of the umbilicus and two in the right upper quadrant. The colon transversum is lifted and the small intestine is followed until the ligament of Treitz is identified. Approximately 10 to 20 cm distal to the ligament of Treitz the jejunum is marked in order to identify the afferent and efferent part. Thereafter, the opening at the umbilicus is enlarged and the jejunum is pulled out. The jejunum is transected using a stapler. Approximately 10–15 cm on the efferent loop an end-to-side anastomosis is made using PDS-4–0 sutures. After determining the jejunostomy site, a gastrostomy tube is carefully pulled through the abdominal wall by using a clamp and brought outside the umbilical opening. The tube is inserted in the Roux-limb after which the balloon is insufflated with a few milliliter. By pulling the catheter, the Roux-limb is pulled against the abdominal wall. After creating a pneumoperitoneum, the Roux-limb is fixed against the abdominal wall with two stitches. After 6 weeks, the tube is changed for a button (Fig. 1).

### Data collection

Data were retrospectively gathered by one of the authors using a predefined data extraction form. The data were extracted from patients electronical medical record system and consisted of baseline characteristics (weight, age, gender, the use of antacids or anti-emetics, underlying conditions or earlier surgery related to delayed gastric emptying, method of feeding prior to LRFJ, follow-up (months), duration of surgery (minutes), and duration of hospitalization (days)) and outcomes (complications, time between surgery, the start of tube feeding after surgery and parental satisfaction).

### Outcomes

Primary outcomes were complications (number of patients with complications and the total amount of complications). Complications, both surgical and maintenance complications (such as leakage, hypergranulation, infection, obstruction, an extraordinary amount of post-operative pain or emesis, and dislodgement/displacement), were divided into early (< 30 days post-operative) and late (> 30 days post-operative). Severity of the complications was rated using the Clavien-Dindo classification, which is a classification used to rank complications in an objective and reproducible manner based on the therapy needed to correct them [22]. Secondary outcomes were parental satisfaction and the time (days) between surgery and the start of tube feeding after surgery. Parental satisfaction was derived from recorded conversations between doctor, parents and, where possible, patients.

### Statistical analysis

Due to the nature of this study only descriptive measurements were used.

## Results

### Baseline characteristics

In this time period, 12 patients underwent a LRFJ in our tertiary referral center. Baseline characteristics of the patients are shown in Table 1. As shown, the age of the patients ranged from 0–17 years old, most of them (*n* = 11) received either proton pump inhibitors and/or prokinetic agents and about half of them were neurologically impaired. Some patients underwent additional imaging studies to objectify the delayed gastric emptying. Contrast studies were performed in three patients with in only one patient demonstrating delayed gastric emptying. In 7/12 patients, a formal gastric emptying test was performed to objectify the delayed gastric emptying. In 5/7, the delayed gastric emptying was confirmed, in one patient, results were unclear, and in one patient, results were normal. Nevertheless, all patients underwent LRFJ due to clinical reasons (deterioration, malnutrition, and idiopathic persistent vomiting).

**Table 1:** Patient characteristics

P-number	Gender	Age (y)	Weight at surgery (kg)	PPI’s pre-LRFJ	Prokinetic agents pre-LRFJ	Underlying condition and earlier interventions	Pre-LRFJ method of enteral feeding	Follow-up (m)
1	F	2	12	No	No	Noonan syndrome, neurofibromatosis-1, gastropexy, pyloric stenosis	Nasoduodenal tube	114
2	M	3	12	No	Yes	Gastroparesis, botulinum injections	Nasoduodenal tube	110
3	M	2	11	Yes	Yes	Gastroparesis, gastropexy	Nasoduodenal tube	109
4	M	8	24	Yes	No	Currarino syndrome, colectomy, ileostomy	Nasoduodenal tube	75
5	F	14	41	Yes	No	Intractable refractory constipation	PEG tube and PEG-J- tube	75
6^a^	F	4	13	Yes	No	Congenital CMV-infection with cerebral abnormalities	Nasoduodenal tube	73
7^a^	M	0	7	Yes	Yes	Bainbridge-Ropers syndrome, botulinum injections	Nasoduodenal tube	24
8^a^	F	17	40	Yes	No	Lissencephaly	Nasoduodenal tube & PEG-tube	16
9^a^	M	12	35	Yes	Yes	Mitochondrial myopathy, TLK2-gene mutation	Nasoduodenal tube and PEG-tube	14
10^a^	M	9	23	Yes	No	Mitochondrial complex I and IV deficiency	PEG-tube	6
11^a^	F	2	9	Yes	Yes	MRFACD	Nasoduodenal tube	5
12^a^	M	11	26	Yes	Yes	Pelizaeus-Merzbacher disease	Nasoduodenal tube	2

*F* female, *kg* kilogram, *M* male, *m* month, *MRFACD* mental retardation and distinctive facial features with or without cardiac defects, *P-number* patient number, *PPI* proton pump inhibitor, *y* year

^a^Neurologically impaired

In addition to the standard measures to improve gastric emptying (feeding and pharmacological interventions), we performed gastropexy in two patients and treated two patients with pyloric botulinum injections. None of the patients had fundoplication before. Four patients received enteral feeding using a PEG(-J)-tube prior to LRFJ, and all other patients received enteral feeding using a nasoduodenal tube prior to LRFJ.

### Outcomes

Complications, details about the surgery, follow-up, and hospitalization can be found in Table 2. A total of 16 complications were noted in 8/12 patients (67%). Most of the complications occurred in the early postoperative period. Severity classified by Clavien-Dindo was grade I (*n* = 13), grade II (*n* = 1), and grade IIIB (*n* = 2). Hypergranulation was treated with silver nitrate sticks. Leakage was often self-limiting or treated by switching to buttons with a shorter length. One patient developed an incisional hernia for which surgery was needed.

**Table 2:** Complications and surgical information

P-number	Hospitalization (d)	Combined surgery	Surgery time (m)	Early complications (< 30 d post-operative)	Late complications (< 30 d post-operative)	Satisfying result
1	9	No	153	Hypergranulation	-	Yes
2	18	No	177	-	-	Yes
3	9	No	180	-	-	Yes
4	14	No	194	Gastric dilation, paralytic ileus	-	Yes
5	9	No	153	-	Intestinal pseudo-obstruction	No, TPN needed
6	3	No	132	-	*-*	Yes
7	8	No	149	Hypergranulation, leakage of insertion opening, incisional hernia^a^	-	Yes
8	6	No		-	-	Yes
9	5	No		Pain, emesis, leakage of insertion opening	-	Yes
10	17	No		Hypergranulation, pressure ulcers, emesis	-	Yes
11	7	Yes, combined with transmeatal drainage of the middle ear	166	Hypergranulation, wound infection	-	Yes
12	8	No		Hypergranulation	-	Yes

*d* days, *m* minutes, *P-number* patient number

^a^With an indication for surgery

Tube feeding was started on the first day after surgery; however, two patients started with tube feeding on day 3 and 11 after surgery. The patient who started 11 days post-operative with tube feeding showed a gastric dilation and paralytic ileus postoperatively (indicated by persistent, severe vomiting, and nausea, confirmed by an abdominal X-ray). Gastric dilatation and paralytic ileus were seen in this patient before in another hospital, after a replacement of a duodenal tube. Both times, the gastric dilation and paralytic ileus were most likely related to the extensive adhesiolysis and an unknown motility disorder.

In 11 of the 12 patients, it was possible to give continuous enteral feeding without significant problems after treatment. The patient who failed treatment showed the clinical picture of intestinal pseudo-obstruction after surgery and began to show more and more inexplicable symptoms. The patient finally switched from enteral feeding to total parenteral nutrition (TPN). After careful observation, it was noted that the patient corrupted the process of feeding herself and the patient was diagnosed with a factitious disorder. The patient was confronted with this and discharged from the hospital. After rehabilitation, the patient was able to eat normal.

Interviews with both patients and parents/caregivers showed that the LRFJ procedures resulted in satisfaction in 11 of the 12 cases. Parents, caregivers, and patients mentioned the reduced hospital visits and the reduced burden of disease, because the button at the jejunostomy site can be changed at home/an outpatient clinic, as the main benefits of the Roux-en-Y feeding jejunostomy.

## Discussion

In our experience, LRFJ was successful in 11/12 patients with delayed gastric emptying. Although complications occurred in 8/12 patients, most of them were minor complications and parents were satisfied in 11/12 cases.

LRFJ is a rare surgical procedure; and to our knowledge, this is the second study reporting on LRFJ in the pediatric population. This makes it difficult to compare our experiences with others. Our study shows a similar amount of complications compared to the first report on LRFJ in children by Neuman and Phillips who reported about LRFJ in five patients [19]. Only minor complications and a delayed start of jejunal feeding in two patients were found. The delayed start of feeding was due to emesis and pain related to visceral hyperalgesia and idiopathic diarrhea. These outcomes correspond with our experiences.

More publications about open Roux-en-Y jejunostomy are available. Recently, a systematic review comparing these publications was published [20]. Open surgery is associated with a different type (more severe) and a higher rate of complications compared to the LRFJ, as there is a high (6–40%) incidence of wound infections [17, 23, 24] and volvulus (14–25%) [24–26] compared to respectively 8% and 0% in our patient population. Despite this difference, the open jejunostomy showed, as expected, the same complications related to the jejunostomy site as the laparoscopic jejunostomy site: leakage (17–43%) [4, 17, 23] and hypergranulation (no percentages available) [27]. In our patient, population leakage occurred in 17% of the patients. In another study with 11 patients, only long-term outcomes but no complications were described [28].

The incidence of volvulus is probably related to the length of the Roux limb and the kind of surgery (open or laparoscopic). Taylor and Ryckman reported, in a series of 25 open Roux-en-Y jejunostomy procedures, a small bowel volvulus around the Roux limb in 20% of the patients. The patients with a volvulus showed a relatively longer Roux limb compared to the patients without volvulus (18.7 + –7.7 vs 14 + –2.3 cm). In one patient with a volvulus the Roux limb was only 6 cm [25]. In the studies of McCann et al. and Singh et al. no details about Roux limb length were described but all patients with volvulus received open surgery [24, 26]. In our series, the Roux limb was relatively short (10–15 cm). No volvulus or stenosis occurred until now.

It is interesting to see that nearly all patients in our study started jejunal feeding one day postoperatively while patients described in other studies started jejunal feeding around 3–7 days after surgery [19, 23, 28]. No clear explanation was given for this delay. Only one study described good results with early (< 48 h) jejunal feeding in 13 patients just like we found [27].

Factitious disorders remain underreported by surgeons. In complex, unexplained pathology a factitious disorder should always be kept in mind. Therefore, we recommend discussing complex patients, like our patient population, in a multidisciplinary team. If pathology remains unexplained and symptoms do not resolve, careful clinical observation might be the only way to bring a factitious disorder to light.

### Strengths and limitations

Despite good results, our study has its limitations. This is because of the retrospective study nature and the relative small number of patients. The retrospective nature makes it for example difficult to know for certain if every complication is noted. Besides this, our study is a case-series with little to no comparison which makes it difficult to compare outcomes to other studies.

Parental satisfaction was not measured using a validated tool. Although (validated) tools to measure parental satisfaction after hospital admission or regarding certain treatments exist, these tools usually evaluate more than just a certain intervention, evaluating hospital admission time, communication, discharge process, etc., which is not applicable for our study. Other studies measure satisfaction using a simple 5-point Likert-scale, comparable to our method of deriving parental satisfaction from conversations. Using a validated tool, evaluating parental satisfaction regarding just the (long-term) outcome of surgery would have strengthened our results, but such a tool is still not available.

In the literature, no data was provided on parental satisfaction after LRFJ and we could not compare our results to parental satisfaction after open Roux-en-Y jejunostomy/LRFJ in other surgical centers.

In addition, the diagnosis delayed gastric emptying was not clearly defined in our population, but prior to performing LRFJ, patients were discussed within our multidisciplinary team. The role of gastric emptying scans/studies was supportive as the outcomes of the gastric emptying scans/studies differed and did not influence the decision to perform LRFJ on these patients.

## Conclusion

Although minor complications after LRFJ are common, this technique is a safe solution in patients with gastric emptying disorders requiring a definitive method of enteral feeding. Information from this study contributes to the scarce available literature on this topic and can be used to counsel parents and patients.

## Abbreviations

LRFJ: Laparoscopic-assisted Roux-en-Y feeding jejunostomy
PEG-J: Percutaneous endoscopic gastro-jejunal
PDS: Polydioxanone
TPN: Total parenteral nutrition
